# Iatrogenic Amputation of the Distal Third of the Penis During Circumcision: Successful Reimplantation Without Magnification

**DOI:** 10.1155/criu/5856486

**Published:** 2026-04-14

**Authors:** Hassami Sawadogo, Clotaire Alexis Marie Kiemdiba Donega Yaméogo, Abdoul-Karim Paré, Abdoul-Karim Ouattara, Fatao Ouédraogo, Harouna Gnanou, Brahima Kirakoya, Adama Ouattara

**Affiliations:** ^1^ Department of Urology, Dedougou Regional Hospital, Dedougou, Burkina Faso; ^2^ Division of Urology, Yalgado Ouedraogo University Teaching Hospital, Ouagadougou, Burkina Faso; ^3^ Division of Urology, Souro Sanou University Teaching Hospital, Bobo-Dioulasso, Burkina Faso

**Keywords:** case report, circumcision, iatrogenic amputation, microsurgery, penile reimplantation

## Abstract

**Background:**

Traumatic penile amputations, although rare, represent complex urological emergencies. Cases of iatrogenic origin secondary to circumcision are an exceptionally uncommon but serious etiology. The standard of treatment is microsurgery. However, in many contexts, particularly in developing countries, access to microsurgery is limited, leading surgical teams to resort to reimplantation techniques without microvascular anastomosis, with variable results.

**Case Presentation:**

An 8‐year‐old boy was admitted to our department for complete amputation of the distal third of the penis during a circumcision. The amputated segment was preserved in saline solution. Emergency reimplantation was performed within a 3‐h timeframe. The procedure included end‐to‐end urethrorrhaphy over a stent, cavernorrhaphy, and vascular alignment, all without the use of loupes or an operating microscope. The patient was discharged on the 25th postoperative day with a perfectly viable reimplanted segment and a satisfactory aesthetic and functional outcome.

**Conclusions:**

This case demonstrates that penile reimplantation can be successful in the absence of microsurgery in children, through meticulous technique and aggressive postoperative management of edema in settings with limited technological resources. It also serves as an urgent reminder of the absolute necessity to prevent these tragic accidents through the standardization and medicalization of circumcision practices. Long‐term follow‐up is essential to assess the erectile and voiding function of this patient.

## 1. Background

Traumatic penile amputation is a rare but dramatic injury, with profound physical, psychological, and functional implications for the patient. While etiologies in adults often include psychiatric self‐mutilation, industrial accidents, or criminal assaults, the predominant cause in pediatric cases is iatrogenic complications from circumcision, followed by domestic accidents and animal bites [1, 2].

Although circumcision is considered a common procedure, it is not without risks. Serious complications, although rare, include hemorrhage, infection, and in very exceptional cases, partial or total amputation of the glans or penis [1, 3, 4]. The frequency of these iatrogenic accidents is significantly higher when the procedure is performed outside of a standardized medical environment by nonmedical or untrained practitioners [1, 3, 5].

The therapeutic standard for amputation is immediate microsurgical reimplantation, aiming to restore urinary function, sensation, and erectile function by repairing vascular and nervous structures [6, 7]. However, in many contexts, particularly in developing countries, access to microsurgery is limited, leading surgical teams to resort to reimplantation techniques without microvascular anastomosis, with variable results [1, 8, 9].

We report here the case of a successful reimplantation of the distal third of the penis in an 8‐year‐old boy following an iatrogenic amputation during circumcision, successfully performed in the absence of microsurgery. This case allows us to discuss technical alternatives and salvage strategies in resource‐limited settings.

## 2. Case Report

S.W., an 8‐year‐old boy, was admitted as an emergency to our department following an accidental transection of the distal third of the penis during a circumcision performed in a treatment room. After being contacted, we recommended the immediate preservation of the amputated segment in isotonic saline and the patient′s admission to the operating room.

The child was previously healthy with no significant past medical history, no known drug allergies, and no previous surgeries. His family history was noncontributory. The circumcision was performed for traditional reasons. The surgical procedure was performed by a urologist (senior specialist). No microsurgical training had been specifically completed by the operating team.

**Figure 1:**
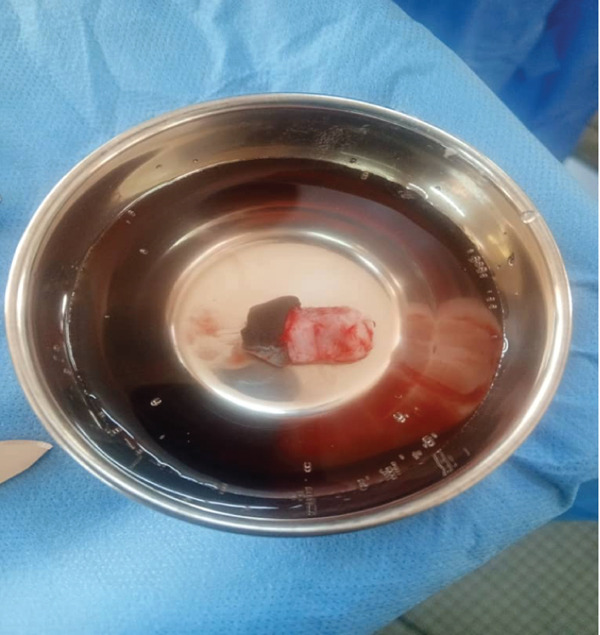
Amputated stump preserved in a container with isotonic saline solution.

On admission, the child was conscious and hemodynamically stable. Clinical examination revealed a complete transection at the distal third of the penis involving the spongy urethra, both corpora cavernosa, and the dorsal neurovascular bundle. The amputated stump had been preserved in a container with isotonic saline solution (Figure 1).

No additional diagnostic imaging was performed preoperatively, as the diagnosis was clinically evident and the urgency of the situation required immediate surgical intervention. The main challenge was the lack of microsurgical equipment, which is not available in our center.

**Figure 2:**
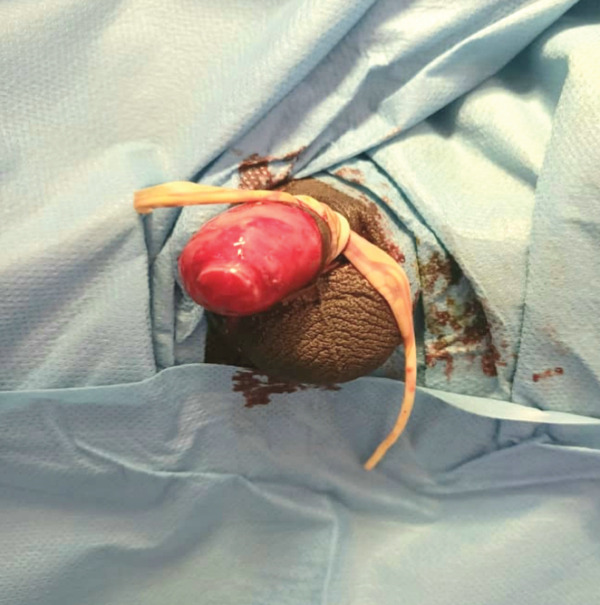
Patient positioning in the operating room.

Surgical intervention was initiated within 3 h after the accident. The total ischemia time was approximately 1 h 15 min, comprising a warm ischemia phase of approximately 15 min (time elapsed between the amputation and immersion of the segment in isotonic saline solution) and a cold ischemia phase of approximately 1 h (time the segment spent preserved in saline solution prior to surgical reimplantation). The entire procedure, from accident to completion of reimplantation, was completed within 3 h. After positioning the patient in the lithotomy position under general anesthesia and applying a sterile surgical drape (Figure 2), the transected structures were identified and prepared. Blood vessels were copiously irrigated with heparinized saline solution to promote their patency.

**Figure 3:**
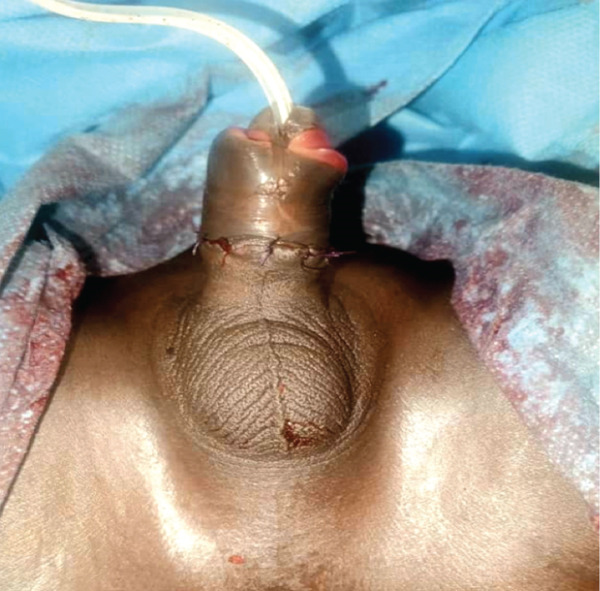
Appearance of the penis at the end of the procedure.

The surgical procedure involved an end‐to‐end anastomotic urethrorrhaphy using 4/0 absorbable suture over a 10 French transurethral catheter; anatomical alignment of the corpora cavernosa, followed by end‐to‐end tunica albuginea repair of the corpora cavernosa with 5/0 nonabsorbable monofilament polypropylene suture; anatomical approximation of the dorsal arteries and veins of the penis with 5/0 absorbable suture (this step consisted of approximation of the periadventitial tissue and fascia of the neurovascular bundle rather than formal intraluminal ligation; the vessel lumens were not ligated. Hemostasis was achieved by direct pressure and suture ligation of visible bleeding points at the transection margin); and circumcision revision to achieve adequate and harmonious skin coverage. The entire reimplantation procedure was performed without magnifying loupes or microsurgical equipment (Figure 3).

Postoperatively, the child received anticoagulant prophylaxis with enoxaparin administered subcutaneously for 14 days. The dose was adjusted to the child′s weight according to pediatric prophylactic recommendations (approximately 0.5 mg/kg/day), in line with current guidelines [10]. This duration was chosen to cover the critical period of vascular remodeling and graft revascularization, estimated at 10–14 days in the literature on composite graft healing. No hematoma formation and no prolonged bleeding from the needle decompression sites were observed during the anticoagulation period, combined with antibiotic prophylaxis with amoxicillin for the same duration, as well as analgesics and daily dressings with petroleum jelly gauze. Needle decompression using a syringe needle was performed every 2 days on the reimplanted segment to reduce inflammatory edema. Technically, a 20‐gauge needle was inserted at a subcutaneous level (not cavernosal) at multiple points along the edematous skin of the reimplanted segment, and gentle aspiration was applied; approximately 0.5–1 mL of serosanguineous fluid was evacuated per session. The procedure was performed under strict aseptic conditions, and no signs of secondary infection were observed. We considered the use of topical heparin bleeding (“chemical leeching”) as an alternative; however, given the limited availability of this technique in our setting and our prior experience with needle aspiration in postoperative penile edema, we opted for mechanical decompression. Medical leeches were not available in our institution. The intravenous line was removed early on the second day, allowing for a switch to oral medication and early mobilization of the patient.

The patient and his family adhered perfectly to the postoperative treatment plan. The interventions were well tolerated, and no adverse or unanticipated events occurred during the hospitalization.

**Figure 4:**
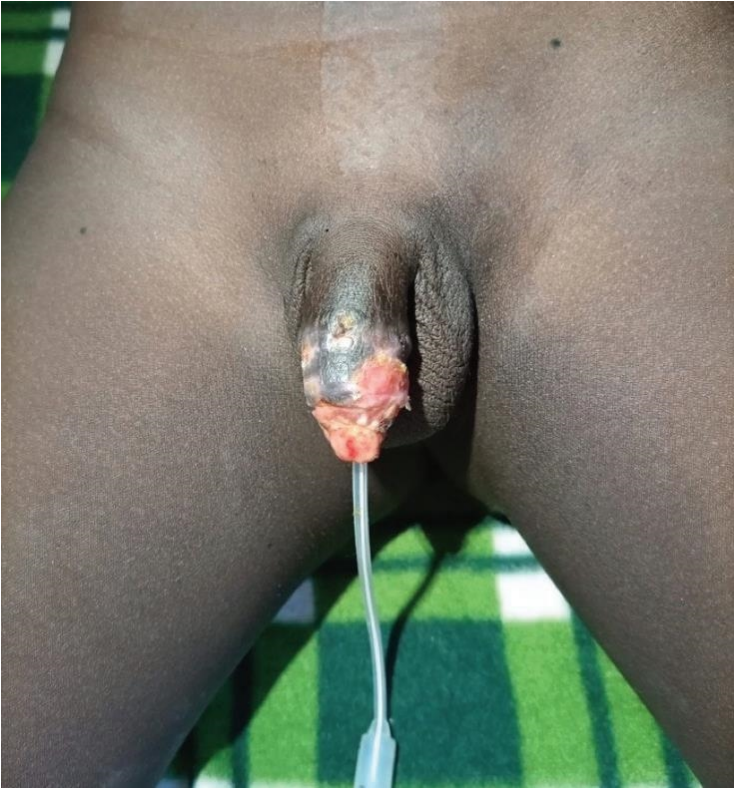
Appearance of the penis at Day 22.

**Figure 5:**
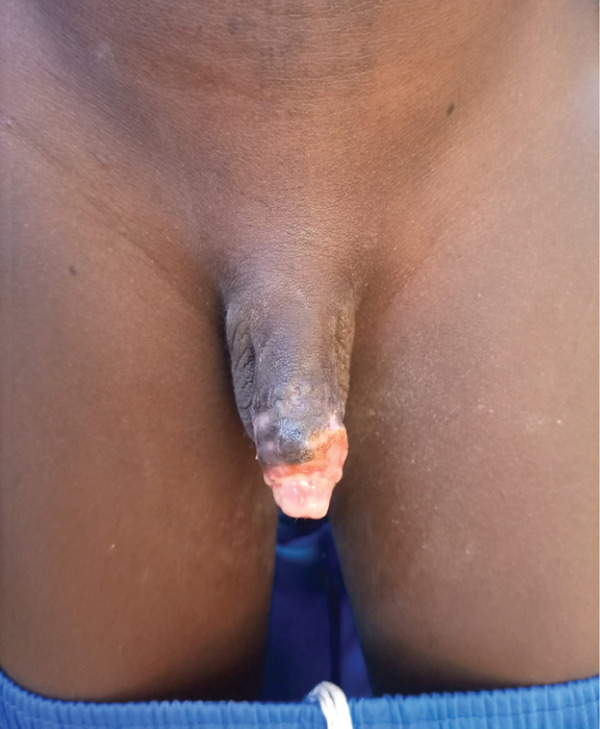
Appearance of the penis at 1 month.

The urinary catheter was left in place for 24 days. Its removal confirmed spontaneous voiding with a normal urinary stream. The patient was discharged on the 25th postoperative day. The outcome was favorable, with a viable reimplanted segment and satisfactory aesthetic and functional results (Figures 4 and 5).

The superficial crust visible on the aspect and glans corresponds to superficial epidermal sloughing. This was not indicative of full‐thickness skin or graft loss; the underlying dermis and subcutaneous tissue remained viable, as confirmed by the favorable evolution observed at 1 month (Figure 5).

**Figure 6:**
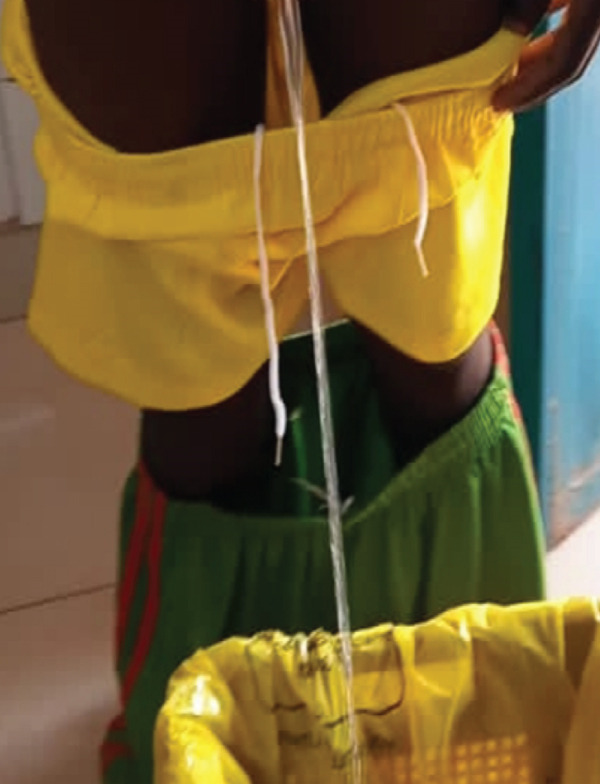
Clinical documentation of the urinary stream at the 6‐month follow‐up visit.

At the 6‐month follow‐up, the family expressed immense relief and gratitude for the successful outcome. The child was able to void normally and was adapting well psychologically. Formal uroflowmetry was not available in our center at the time of follow‐up. Clinical assessment of voiding function was therefore performed by direct observation: The child demonstrated a continuous, well‐calibrated urinary stream without straining, hesitancy, or dribbling. No visible fistula was observed. A photograph of the urinary stream was obtained to document stream caliber objectively (Figure 6). No clinical signs suggestive of urethral stricture were present at this stage, though we acknowledge that long‐term surveillance, including formal urodynamic evaluation, remains essential.

## 3. Discussion

### 3.1. Epidemiology and Iatrogenic Context

Our case fits within the well‐documented but rare context of iatrogenic penile amputations during circumcision. A 2017 retrospective study by Diabaté et al. on penile trauma reported that 60% of cases occur in children aged 12 and under, with circumcision being a significant cause among amputation etiologies [2]. This is corroborated by case series from Diabaté and Kouka and Essid et al., which specifically describe this type of accident [1, 3]. Most of these accidents occur when the procedure is performed outside an operating room by nonmedical or inadequately trained personnel, highlighting the imperative to medicalize this procedure [1, 3, 5].

### 3.2. Surgical Management: A Resource‐Adapted Paradigm

Microsurgical reimplantation with anastomosis of the dorsal arteries, deep dorsal vein, and nerves is considered the gold standard, offering the best results in terms of tissue viability, sensation, and erectile function [6, 7, 11]. However, as emphasized by Babaei and Safarinejad in their systematic review, this technique is not universally available, and its success depends on specialized expertise and equipment [6].

Our case, like those reported by Diabaté and Kouka and Essid et al., demonstrates that reimplantation without microsurgery can be a reliable alternative, especially in children [1, 3]. The success of this approach can be explained by several factors highlighted in the literature:

• The rich collateral vascularization of the penis: Penile vascularization comes from both the cavernosal (deep) and dorsal arteries. Even without microsurgical anastomosis, passive perfusion via the sinusoidal spaces of the corpora cavernosa may be sufficient to maintain the viability of the distal segment, particularly for clean, distal amputations [1, 6, 8]. From a physiological standpoint, the mechanism of survival of the distal segment in nonmicrosurgical reimplantation is best understood as a composite graft phenomenon. In the early postoperative phase (approximately 24–72 h), the graft survives through plasmatic imbibition, whereby oxygen and nutrients diffuse passively from the recipient bed across the anastomotic interface without active vascular flow—a process analogous to skin graft “plasmatic imbibition.” This phase is then followed by inosculation (Days 3–5), during which recipient and graft capillaries realign and establish flow, ultimately leading to full revascularization. In our case, the dorsal arteries were approximated at the fascial level rather than formally anastomosed; therefore, retrograde arterial flow through the anastomosed site is unlikely. The predominant mechanism was likely spongiosal carry‐over through the repaired urethra and re‐established continuity of the corpus spongiosum, supplemented by progressive neovascularization favored by the young age of the patient.
• The patient′s age: Children have an excellent capacity for healing and neovascularization, which favors the take of the composite graft represented by the reimplanted segment [1, 3].
• The preservation of the amputated segment in saline solution.
• The favorable timeframe for management, as the incident occurred in a healthcare center with a responsive medical team.
• The aggressive postoperative strategy: Edema management is a critical point in nonmicrosurgical reimplantations. Skin necrosis secondary to venous congestion is the most frequent complication [6, 8, 9]. The technique of iterative needle decompression (“saignées”), used in our case, is a salvage method aimed at creating artificial venous drainage and preventing necrosis. This maneuver compensates for the absence of venous anastomosis and proved crucial for success.

A limitation of our report is the lack of a detailed preoperative patient history, though it was confirmed that he was otherwise healthy. Furthermore, the absence of microsurgical capabilities was the primary technical challenge.

### 3.3. Results, Complications, and Long‐Term Follow‐Up

Historical series, such as that of McRoberts et al., reporting 18 reimplantations without microsurgery, document a high rate of complications: skin necrosis (12/18), partial glans necrosis (10/18), total necrosis (1/18), and total loss of sensation in the reimplanted segment in all patients [9]. The technique of burying the denuded penis in the scrotum, described by McRoberts et al. and adopted by Bhanganada et al., was an attempt to prevent skin necrosis while awaiting secondary coverage [9].

The results of our case, with a favorable short‐term outcome, are better than those expected based on these older series. This may be explained by the patient′s young age, the cleanliness of the transection, and the effectiveness of postoperative edema management. However, long‐term follow‐up is essential to assess erectile, voiding, and sensory function, which remain problematic even after some microsurgical reimplantations [6, 11]. The frequent loss of sensation is often permanent in the absence of nerve repair [6, 9].

The prognosis for full erectile function remains guarded and will be a key focus of long‐term monitoring.

### 3.4. Psychological Considerations and Prevention

Beyond physical repair, the psychological dimension of this trauma is major, both for the child and his family. As emphasized by Rimtebaye et al. and Odzébé et al. in their series on amputations, multidisciplinary management including psychological support is essential [12, 13]. Prevention remains the cornerstone of managing these accidents. It requires adequate training of practitioners and the systematic performance of circumcision in a secure medical environment, as advocated by Essid et al. and Diabaté and Kouka [1, 3].

## 4. Conclusion

This case illustrates that in a resource‐limited context, penile reimplantation without microsurgery can be considered with a reasonable hope of success, owing to meticulous surgical technique and an aggressive postoperative strategy for edema control. It does not challenge the superiority of microsurgery but offers a realistic salvage paradigm. It also serves as an urgent reminder of the absolute necessity to prevent these tragic accidents through the standardization and medicalization of circumcision practices. Long‐term urological and psychological follow‐up of this patient will be essential to document the full outcome of this case.

## Author Contributions

H.S.: conceptualization, clinical management, and draft and revision of the manuscript. C.A.M.K.D.Y.: literature review and manuscript revision. A‐K.P.: manuscript revision and critical review. A‐K.O.: clinical management and manuscript revision. F.O.: clinical follow‐up and manuscript revision. H.G.: surgical assistance and data collection. B.K.: critical revision of the manuscript. A.O.: supervision and critical revision. H.S. (corresponding author and manuscript guarantor) had full access to all of the data in this study and takes complete responsibility for the integrity of the data and the accuracy of the data analysis.

## Funding

No funding was received for this manuscript.

## Disclosure

All authors have read and approved the final version of the manuscript.

## Consent

Written informed consent was obtained from the patient′s guardian for the publication of this case report and accompanying images.

## Conflicts of Interest

The authors declare no conflicts of interest.

## Data Availability

The authors have nothing to report.
